# Occupational risk factors for surgically treated lumbar disc herniation – a 33-year follow-up

**DOI:** 10.5271/sjweh.4253

**Published:** 2025-11-01

**Authors:** Jens Wahlström, Per Liv, Albin Stjernbrandt, Arkan S Sayed-Noor, Sebastian Mukka, Charlotte Lewis, Jennie A Jackson

**Affiliations:** 1 Department of Epidemiology and Global Health, Umeå University, Umeå, Sweden.; 2 Department of Public Health and Clinical Medicine, Umeå University, Umeå, Sweden.; 3 Clinical Sciences Department, College of Medicine, University of Sharjah, Sharjah, United Arab Emirates.; 4 Department of Diagnostics and Intervention (Orthopaedics), Umeå University, Umeå, Sweden.; 5 Department of Occupational Health Sciences and Psychology, University of Gävle, Gävle, Sweden.

**Keywords:** disability, early retirement, heavy lifting, job exposure matrix, low back pain, manual material handling

## Abstract

**Objectives:**

This study aimed to assess the associations between occupational biomechanical factors and occurrence of surgically treated lumbar disc herniation (LDH) and describe the consequences in terms of early exit from the labor market.

**Methods:**

A cohort of 262 850 male construction workers participating in a national occupational health surveillance program was followed prospectively for 33 years (1987–2019). Occupational biomechanical exposures were assessed by a job exposure matrix (JEM) based on specific occupational groups. Workers who underwent surgical treatment for LDH were identified from the national patient register and data on disability pension from the Swedish Social Insurance Agency. Poisson regression models were used to estimate relative risks (RR) and 95% confidence intervals (CI) for biomechanical exposures, adjusted for age, body mass index, smoking status, height and time period.

**Results:**

There were 2451 cases of surgical treatment for LDH and the incidence peaked at age 40–45 years. Increased risks were found for often lifting >25 kg (RR 1.77, 95% CI 1.06–2.94), extreme lumbar flexion/extension (RR 1.60, 95% CI 1.37–1.88) and high exposure to whole-body vibration (RR 1.32, 95% CI 1.05–1.65). Among cases, the mean age for exiting the labor market due to disability pension was 55.9 years for white-collar workers and 51.7 years for construction workers.

**Conclusions:**

Occupational exposure to heavy lifting and working in non-neutral back postures was associated with increased risk of surgical treatment for LDH. Construction workers who have had surgery for LDH exited the labor market with disability pension earlier than white-collar workers.

Low-back pain (LBP) is a leading cause of disability globally (1–3). In 2020, the global prevalence of LBP was 619 million cases, and the prevalence projection for 2050 was for 843 million cases (4). The lifetime prevalence of LBP has been estimated at 39% (5). LBP can result in considerable personal suffering and decreased quality of life as well as high societal costs due to reduced productivity, work absenteeism, and healthcare expenditures (6).

LBP is often unspecific, but sometimes the cause of the symptoms is known, for example in sciatica due to lumbar disc herniation (LDH). Also known as a slipped or ruptured disc, LDH is a condition where the gel-like center of the intervertebral disc, the nucleus pulposus, pushes against the fibrous outer rings, annulus fibrosus, of the disc. This can lead to disc bulging or rupture, which can put pressure on the nearby spinal nerves by direct mechanical compression and/or due to the resulting local inflammation. Symptoms include local pain that can radiate down the leg and foot, and numbness or tingling can occur in the innervated area of the affected nerve which can result in weakness of the muscles innervated by the affected nerve. LDH are most common at the lowest vertebral levels L4-L5 and L5-S1 and occur most commonly in mid-life, peaking at around age 40–45 years (7). International guidelines recommend that treatment starts with physiotherapy and medication (non-operative treatment) followed by surgery when non-operative treatment has failed or when major radicular weakness is present (8). A recent meta-analysis suggested that discectomy is superior to non-operative treatment in reducing leg pain and disability (9), however the effects were rather small. Better surgical outcomes have also been reported among younger (ie, adolescents) compared to older patients (10).

Generally, there is less known about risk factors associated with LDH than LBP. However, both individual risk factors, such as body height (higher risk for taller individuals), high body mass index (BMI) (11–13), smoking (6, 14), heredity (15–17), and occupational risk factors, such as heavy work, lifting and working in twisted and bent positions have been associated with an increased risk of LDH (6, 11, 18–21). In addition, occupational exposure to whole-body vibration (WBV) has been associated with increased risk in several studies (6, 22, 23), though a recent systematic review found insufficient evidence for professional driving as a risk factor for LDH (21).

Evidence on whether occupational biomechanical factors increase risk for operative treatment of LDH is scarce, though there are previous studies showing associations between occupational biomechanical exposures and hospitalization due to LDH (11, 18, 24). Further, knowledge is lacking on the long-term consequences of operative treatment for LDH, for example, on labor market re-entry, work ability, or rates of disability pension or early retirement. This knowledge is required to understand the full consequences of lumbar disorders among working-age subjects.

This study aimed to (i) assess the association between occupational biomechanical factors and occurrence of surgically treated LDH and (ii) describe the consequences in terms of early exit from the labor market.

## Methods

A large cohort of male construction workers was followed prospectively for 33 years (1987–2019) to identify operative treatment for LDH and its association with prior occupational physical exposures. The Regional Ethical Review Board in Umeå approved the study (2017/16-31).

### Study cohort

The study cohort was selected from a total of 389 132 Swedish construction workers who participated in health examinations as part of a national health surveillance program conducted from the late 1960s until 1993 and comprise the Bygghälsan register. While participation was voluntary, ≥80% of eligible workers completed at least one health examination (25).

### Individual factors

Worker height, weight, age, smoking status, and specific trade (‘job title’) were recorded on examination. BMI was calculated as weight/(height^2^). Worker smoking status was categorized as ever, never and unknown.

### Occupational biomechanical exposure and job exposure matrix

Worker job titles were mapped into 21 occupational groups defined by occupational health service experts at the time of the surveillance programs. Occupational groups comprised jobs with similar tasks and workers with similar background training. There was also a group for unclassifiable jobs. Full details of the job-to-group mappings have been published previously (26).

Occupational biomechanical exposures were assigned to each occupational group using a job exposure matrix (JEM) developed for use with the Construction Worker Cohort (*Bygghälsokohorten* in Swedish). The JEM used in the current study contained four biomechanical exposure factors that were each rated on a 3-point scale ranging from 1 (low) to 3 (high) (table 1). Two experts independently rated the average exposure intensity or frequency experienced by a worker over a working day for all occupational groups (except ‘other’) based on ergonomic assessments conducted in the 1970’s for each job title (26). All ratings were done blinded to the number of cases in each occupational group. Ratings were compared and discussed by the experts to resolve any disagreements. A similar process was performed by a single expert to determine WBV exposure, rated on a 3-point scale ranging from none (1) to high (3). The reference group comprised foremen and white-collar workers.

Using the job title reported on the last health examination, JEM exposures for the corresponding occupational group were assigned to each worker. Any changes in job title during follow-up were not accounted for in the analyses. JEM ratings for all exposure variables and occupational groups are presented in the supplementary material, www.sjweh.fi/article/4253, table S1.

**Table 1:** Job exposure matrix of biomechanical risk factors. Assigned ratings reflect average daily exposure levels across all workers and all jobs and tasks for each occupational group.

Exposure	Rating
Frequency of heavy back loading	1 – 3 ^a^
Frequency of lifting more than 25 kg	1 – 3 ^a^
Frequency of static, non-neutral postures	1 – 3 ^a^
Frequency of extreme trunk postures (flexion or extension)	1 – 3 ^a^
Whole body vibration exposure	1 – 3 ^b^

^a^ 1 = low, 2 = moderate, 3 = high ^b^ 1 = none, 2 = acceptable, 3 = high

### Self-reported exposure and low-back pain

A sub-group of the study cohort answered a questionnaire (during health examinations conducted 1989–1993) which included self-reported frequency of exposure to heavy lifting, forward bending or twisted working postures, and LBP in the previous 12 months (each rated on a 5-point scale). From the study cohort, 69 489 workers responded to at least one of these additional questionnaires. We used this sub-group with self-reported individual-level exposure assessment data as complementary data to the JEM-based occupational group-level exposure analyses conducted in the full study cohort, as detailed below.

### Outcomes

*Lumbar Disc Herniation.* Data on surgery due to LDH were collected from a linkage with the Swedish National Inpatient Register. This register contains information from 1 January 1987 onwards, and includes codes for diagnoses and operative procedures. We included data up to 31 December 2019. To be classified as a case with LDH, the worker had to be registered with both a relevant main diagnosis and a surgical procedure. To be a case during the period 1987–1996, individuals were required to have the ICD-9 code 722.1 and any of the surgical codes 8340, 8341 or 8349. To be a case during the period 1997–2019, individuals were required to have the ICD-10 code M51.1 and any of the operative codes ABC07, ABC26, ABC36, ABC56 or ABC66. Workers could also have other diagnoses and/operative codes at admission in addition to the codes required by the case definition.

In a comparative analysis (see the supplementary material), we investigated a case definition based only on hospitalization due to a relevant main diagnosis (ICD-9 722.1 and ICD-10 M51.1), but did not require surgical treatment.

### Early retirement / disability pension

Swedish Social Insurance Agency registry data were obtained for the full follow-up period to investigate labor market exit due to early retirement via disability pension. In Sweden, disability pension can be part- or full-time. We defined early retirement as ≥25% time on disability pension to have a sensitive metric to assess long-term impaired work ability as well as to align with previous studies using Swedish registers (27). There has been substantial variation in the eligibility criteria of disability pension during the observation time, and generally it has become harder to receive this benefit.

### Exclusion criteria

**Figure 1:**
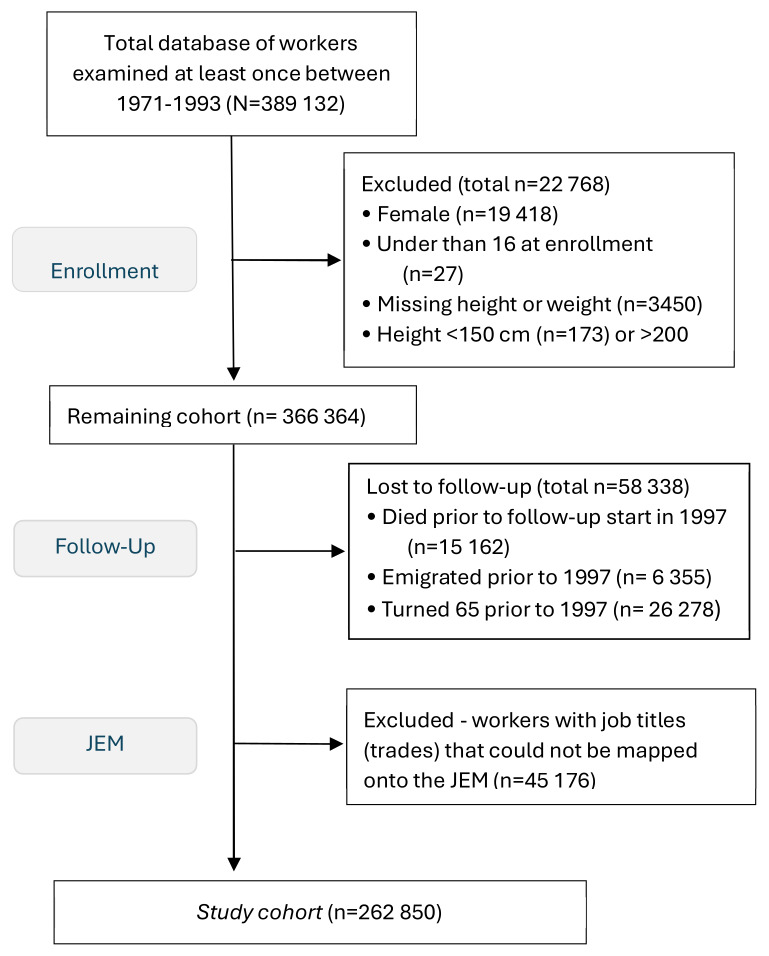
Exclusion flow chart from total database to construction worker study cohort. [JEM=job exposure matrix.]

Female workers were excluded since they comprised only 5% of the population and most belonged to the ‘other’ occupational group with unclassifiable jobs. Workers were also excluded who were: <16 years at first health examination; unusually short (<150 cm) or tall (>200 cm); lacked height or weight data; or had died or emigrated prior to the start of the follow-up period. To focus on etiology in working-age, workers who turned 65 prior to the start of the observation period were excluded from follow-up. Workers for whom no job title was recorded in any medical examination or who belonged to the group with unclassifiable jobs were removed since they could not be mapped onto the JEM. The remaining 262 850 workers comprised the study cohort (figure 1).

### Analyses

Linkage between the Construction Worker Cohort, the Swedish National Inpatient Register, and the Swedish Social Insurance Agency Register was conducted using the unique personal identity number assigned to Swedish residents.

Incidence rates (IR) of surgically treated LDH were calculated for the study cohort over the follow-up period 1987–2019 (figure 1). Person-years were summed from the start of follow-up (1 January 1987) until the earliest occurrence of any the following endpoint criteria: surgical treatment for LDH, censoring for death or emigration, the year the worker was >65 years of age, or until the end of the observation period (31 December 2019).

Robust Poisson regression models were used to estimate relative risks (IR ratios) and 95% confidence intervals (CI) for each biomechanical factor. Follow-up time was split into one-year intervals, permitting separate modelling of the effect of the calendar time and age, under the assumption of a constant year-wise IR (28). Adjustments were made for age, BMI, height, calendar times (continuous variables) and smoking status. The continuous variables were modelled using restricted cubic splines with three knots placed at the 10^th^, 50^th^ and 90^th^ percentiles. In all models, white-collar workers and foremen were used as the reference group. P-values and CI were obtained from robust Hubert-White standard errors. This procedure was also repeated for the sub-group of the cohort for whom self-reported data on frequency of heavy lifting, forward bending or twisted working posture and low back pain were available. In a separate robust Poisson model, IR of surgically treated LDH were modelled using only calendar time and age as independent variables. Further, crude associations between surgical treatment and individual factors (age, BMI, smoking status, height) were estimated using robust Poisson regression.

Analyses of disability pension were descriptive and reported group-level frequencies and mean ages.

All analyses were performed using R v4.0.3 (29). Poisson models were fitted using the Glm function in the rms package (30).

For comparison, we performed additional analyses with a case definition that only required hospitalization for a relevant main diagnosis (ICD-9 722.1 and ICD-10 M51.1) without demand for operative treatment followed the same procedure described above for the primary outcome of surgically treated LDH.

## Results

**Figure 2:**
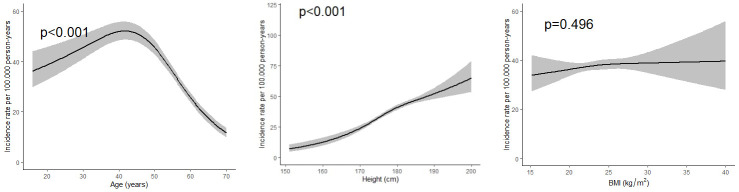
Individual factors (modelled as continuous variables) and relative risk for surgery due to lumbar disc herniationLDH (N=2451) from crude models: (a) age – shown in periods, each one-third of the follow-up period, (b) height and (c) body mass index (BMI).

A total of 6 080 413 person-years of observation were accumulated during follow-up (1987–2019) among the 262 850 male construction workers in the total study cohort (figure 1). There were 2451 cases of surgically treatment for LDH, which resulted in a 33-year IR of 40.3 cases per 100 000 person-years (95% CI 38.7–41.9). IR varied slightly over time between different age groups (data not shown), but the incidence consistently peaked at age 40–45 years (figure 2a). An increased risk was observed with increased height (figure 2b), but not with increased BMI (figure 2c). Being a current smoker at the time of the health examination was associated with an increased risk (RR 1.15, 95% CI 1.06–1.26) compared to those who had never smoked. Neither former smokers nor workers with unknown smoking status had statistically significantly increased risks.

### JEM-based occupational exposure and occupational group

Frequency of lifting >25 kg was the occupational biomechanical exposure variable with the highest relative risk (RR 1.77, 95% CI 1.06–2.94) for the highly exposed group, with a pattern of an exposure-response association (table 2). Work in extreme flexion or extension and work in static non-neutral postures were both associated with an approximately 60% increased risk for the highest exposed group (table 2). A high exposure to WBV was also associated with an increased risk, but there was a lack of an exposure–response association (table 2).

**Table 2:** Biomechanical exposure factors and relative risks for surgically treated lumbar disc herniation (LDH) (N=262 850, including 2451 cases) among male construction workers. Poisson regression models adjusted for age, height, body mass index, smoking and calender time. JEM terms, except whole-body vibration, are for frequency of time spent working in the listed postures or under the listed loads. [N=number of workers; IR=incidence rate per 100 000 person-years; RR=relative risk; CI= confidence interval]

		N	Person-years	Cases	IR	RR	95% CI
Heavy back loading							
	Reference	34 614	698 512.5	208	29.78	1.00	1
	Low	12 866	265 549.6	120	45.19	1.49	1.19–1.87
	Moderate	175 774	4 318 094.9	1748	40.48	1.30	1.13–1.51
	High	39 596	798 256.2	375	46.98	1.54	1.30–1.82
Lifting more than 25 kg							
	Reference	34 614	698 512.5	208	29.78	1.00	1
	Low	52 840	1 301 145.7	463	35.58	1.15	0.97–1.35
	Moderate	174 195	4 051 827.5	1764	43.54	1.42	1.23–1.64
	High	1201	28 927.6	16	55.31	1.77	1.06–2.94
Static, non-neutral trunk posture							
	Reference	34 614	698 512.5	208	29.78	1.00	1
	Low	22 805	485 598.3	194	39.95	1.30	1.06–1.58
	Moderate	199 320	4 744 701.3	1973	41.58	1.35	1.17–1.56
	High	6111	151 601.2	76	50.13	1.62	1.25–2.11
Extreme trunk postures (flexion or extension)							
	Reference	34 614	698 512.5	208	29.78	1.00	1
	Low	20 325	423 402.3	174	41.10	1.34	1.10–1.65
	Moderate	144 191	3 577 028.5	1390	38.86	1.25	1.08–1.45
	High	63 720	1 381 469.9	679	49.15	1.60	1.37–1.88
Whole-body vibration							
	Reference	34 614	698 512.5	208	29.78	1.00	1
	Low	201 081	4 795 936.7	2012	41.95	1.36	1.18–1.58
	Moderate	13 686	294 726.6	112	38.00	1.22	0.97–1.54
	High	13 469	291 237.5	119	40.86	1.32	1.05–1.65

Most occupational groups showed an increased risk of surgery for LDH compared with the reference group (supplementary table S2) with highest risks found for (descending order): crane operators, roofers, refrigerator technicians, plumbers, concrete workers and floor layers (RR 1.81–1.54).

### Self-reported exposure and low-back pain

There were 591 cases in the subgroup of workers from whom self-reported exposure and pain data were obtained (N=69 489 workers; 30% of the study cohort). Both frequency of heavy lifting and working in bent or twisted postures showed exposure–response associations with surgery for LDH (table 3). The RR for the highest exposed groups were very similar to those seen in the total cohort in the JEM-based analyses. Frequency of self-reported LBP in the previous 12 months were associated with surgery in a dose–response pattern with workers reporting LBP often having a RR of 4.76 (95% CI 3.59–6.30) (table 3).

**Table 3:** Self-reported exposure and pain ratings and the relative risk for surgically treated lumbar disc herniation in the sub-group of the study cohort (N=69 489 including 591 cases) who provided self-reported exposure and/or pain level ratings for at least one of the following questions on health examination (1989–1993). Poisson regression models adjusted for age, height, body mass index, smoking and date of surgery. [N=number of workers; IR=incidence rate per 100 000 person-years; RR=relative risk; CI=confidence interval]

		N	Person-years	Cases	IR	RR	95% CI
Frequency of heavy lifting							
	Rarely	9496	223 987.0	51	22.77	1.00	1
	Quite rarely	5656	146 378.1	42	28.69	1.19	0.79–1.79
	Sometimes	23 725	628 128.2	171	27.22	1.11	0.81–1.51
	Fairly often	18 870	515 224.4	188	36.49	1.43	1.05–1.91
	Often	11 742	319 250.9	139	43.54	1.68	1.22–2.32
Frequency of forward-bending or twisted working postures							
	Rarely	8562	205 441.5	45	21.90	1.00	1
	Quite rarely	5410	144 812.6	39	26.93	1.14	0.74–1.76
	Sometimes	17 953	480 898.7	138	28.70	1.21	0.87–1.70
	Fairly often	20 024	537 411.5	191	35.54	1.47	1.06–2.04
	Often	17 534	463 307.0	179	38.64	1.60	1.15–2.22
Frequency of low-back pain during the last 12 months							
	Rarely	16 286	444 716.6	78	17.54	1.00	1
	Quite rarely	10 345	276 974.0	60	21.66	1.24	0.89–1.74
	Sometimes	24 716	651 817.2	173	26.54	1.53	1.17–1.99
	Fairly often	10 866	279 693.0	145	51.84	2.98	2.26–3.93
	Often	6915	166 922.9	130	77.88	4.76	3.59–6.30

### Disability pension

In total, 13.6% (N=35 671) of the study cohort received disability pension (at a minimum rate of 25%). Among surgical cases, the rate was nearly double (25.1%) with a total of 614 cases receiving disability pension. Further, the mean age of retirement onset was lower among cases (51.7 years) than non-cases (56.4 years). When stratifying by occupational class among the surgical cases, the mean age for onset of disability pension was 55.9 years for white-collar cases and 51.7 years for construction worker cases.

### Comparative analyses

In total, 3172 workers were hospitalized with a relevant LDH diagnosis, and the overall IR was 52.3 per 100 000 person-years (95% CI 50.5–54.1).

The same patterns described above for the surgical case requirement were also observed when using this case definition (supplementary tables S2 and S3).

## Discussion

To our knowledge, this is the largest prospective cohort study to date to assess occupational biomechanical risk factors for operative treated LDH. We found evidence that occupational biomechanical factors were associated with increased risk for surgery among male construction workers. Specifically, increased risks were observed with increased frequency in occupational exposure to heavy back loading, lifting >25 kg, static non-neutral working postures, extreme lumbar flexion or extension and level of WBV based on JEM-based exposure assessment. In a sub-group of the cohort where additional data were available, increased risk was also observed for increased frequency of self-reported heavy lifting and working in bent or twisted postures. The highest effect estimate was observed for frequency of self-reported LBP in the last 12 months. The rate of early retirement due to disability pension (at a minimum rate of 25%) was nearly twice as high for cases as for the total study cohort (25.1% versus 13.6%), and the mean age for retirement for construction worker who underwent surgery was lower than for white-collar workers who had surgery (51.7 years versus 55.9 years).

High risk estimates were observed for workers who had reported symptoms in the low back (ie, LBP) during the year preceding the health examination. The RR were also higher (11.6) when we performed a stratified analysis and only studied the cohort during the ICD-9 classification era (1987–1996; data not shown), indicating that the association between self-reported symptoms and surgery of LDH declines over time. In the same cohort, an increased risk for surgical treatment of neck spondylosis was observed for workers who reported pain (31). These results stress the need for more knowledge on the pathways from early and mild self-reported symptoms to more severe conditions. One important question to answer is whether disease progression can be influenced by treating both the symptoms and the exposure by modifying occupational biomechanical exposures.

Among individuals who underwent operative treatment for LDH, construction workers exited the labor market earlier than white-collar workers. This may reflect decreased work ability in jobs with high biomechanical exposures. Previous studies have shown that physically demanding jobs increase the risk of disability pension, however those studies have not studied specific diagnoses prior to exiting the labor market but instead focused on associations between occupational exposure and subsequent disability pension (27, 32). Our findings suggest that future research could try to capture the whole trajectory — from occupational exposure to diagnosis to labor market exit.

In this study, we used operative treatment as a proxy for a conclusive diagnosis for LDH. Given that surgery is typically reserved for cases presenting with serious pain or progressive neurological deficits, the definition is also a proxy for the severity of symptoms. Our case definition is unlikely to have included asymptomatic LDH cases and did not include workers with LDH who were not treated operatively; thus, it is likely to have underestimated both the prevalence of LDH and the level of risk associated with biomechanical exposures and LDH. However, risk patterns were similar when only using hospitalization for LDH as case definition (supplementary tables S2 and S3) to those when operative treatment was required. Further, the number of cases were reasonably similar between the two different case definitions – 2451 cases having surgery versus 3172 being hospitalized – which shows that hospital admission for LDH often results in surgical treatment. There is a possibility for misclassification of diagnosis or surgical treatment in the national patient register, however this would likely be random with respect to occupational exposure. Further, the validity of the register is high, especially regarding more severe diseases (33). The long follow-up time is a strength, however, there has been an extensive development in surgical procedures since the 1980s, which may have influenced the likelihood of LDH surgery over time. We have tried to account for this by adjusting our models for year of surgery (time period variable).

A JEM approach was used in the total study cohort to assign occupational biomechanical exposures based on job title. Two raters independently evaluated historical records from ergonomists who had detailed information about biomechanical exposures and tasks for each job title. This approach has been deemed the best available method for retrospective exposure assessment in cohort studies (34). The JEM approach was selected given that the original exposure evaluations were insufficient to facilitate comparison of specific biomechanical factors, such as specific trunk postures, frequency of heavy lifting or periods in non-neutral or extreme trunk postures. The approach has its limitations since several of the exposure factors in the JEM assessed overlapping aspects of biomechanical exposure aspects (eg, frequency of static non-neutral postures and frequency of extreme trunk postures), and thus some factors were highly correlated. It is therefore difficult to isolate the influence of individual factors. The results must therefore be viewed as proxies for complex exposure patterns and should not be interpreted simply as individual exposure–response associations for a given factor. In addition, the JEM ratings were made at the occupational group level (based on job title) and thus did not account for individual differences in work strategies, anthropometrics or task assignments within a job title. However, we did assess individual ratings of both heavy lifting and working in bent or twisted positions available from about a third of the cohort and observed the same exposure-response patterns as for the JEM-based analyses which gives credibility to both our JEM and our results.

Both JEM-based and self-rated exposures lack specificity for cumulative exposure or latency from first exposure to the date of surgery. Further, no consideration or adjustment was made for potential changes in job title after the first health examination. In Sweden, a large proportion of construction employees are skilled workers with relatively high income compared to other blue-collar workers, so workers tend to stay within the construction industry. Further, it is unlikely that workers changed between branches within the industry (eg, from plumber to carpenter). However, it is plausible that some workers changed job tasks and titles. It is also likely that the jobs themselves changed over time due to changes in technology and working methods. Any such changes were not reflected in the JEM approach to exposure assignment. Further, workers who were sensitive to LDH risk factors or experienced LBP may have exited the construction industry, which would result in a so-called healthy worker survivor bias (35, 36). All the limitations above would tend towards an attenuated estimate of risk, and any resulting overestimation in person-time of observation would lead to underestimation of disease rates. Finally, in the statistical analysis, adjustments were made for potential confounders (where data were available), but the direction and magnitude of residual confounding is difficult to assess.

We have previously found that jobs with high biomechanical demands in this cohort were associated with an increased risk of hospitalization for LDH (11). In the present study, we have used both a JEM and subjective ratings to assess exposure and used a stricter case definition (ie, hospitalization plus surgical treatment of LDH), and we observed increased RR for several biomechanical exposure variables. Kuijer et al's systematic review and meta-analyses (21) concluded that there is moderate-to-high-quality evidence that LDH can be classiﬁed as a work-related disease depending on the level of exposure to bending or twisting of the trunk and lifting and carrying in physically demanding work with meta-RR of about 2.5. Our result further supports these associations, although the RR observed in our study were generally lower than Kuijer and colleagues' reported meta-RR (21). In their systematic review, professional driving (ie, WBV) was not statistically associated with LDH, and our JEM-based results were borderline significant with RR around 1.30 and no clear exposure–response association. The occupational group of drivers had a RR of 1.07 (95% CI 0.67–1.70) compared to white-collar workers (supplementary table S1). Thus, more studies are needed to clarify in what way WBV influence the risk of LDH.

### Concluding remarks

Occupational exposure to heavy lifting and working in non-neutral back postures was associated with increased risk of surgical treatment for LDH. Construction workers who have had surgery for LDH exited the labor market with disability pension earlier than white-collar workers.

## Supplementary material

Supplementary material
