# Supplementary material for: Occupational risk factors for surgically treated lumbar disc herniation – a 33-year follow-up
Source: Scand J Work Environ Health. 2025 Oct 30;51(6):550–8. doi: 10.5271/sjweh.4253 (PMC12593708; doi:10.5271/sjweh.4253)
Supplement: Supplementary material [file SJWEH-51-550-S001.pdf]

Occupational risk factors for surgically treated lumbar disc herniation – a 33-year follow-up[1]

by Jens Wahlström, PhD[2] *Per Liv, PhD, Albin Stjernbrandt, PhD, Arkan S Sayed-Noor, PhD, Sebastian Mukka, PhD, Charlotte Lewis, PhD, Jennie A Jackson, PhD*

1. Supplementary material
2. Correspondence to: Jens Wahlström, Department of Epidemiology and Global Health, Umeå University, Umeå, Sweden. [E-mail: jens.wahlstrom@umu.se].

Table S1. Job-exposure matrix (JEM) ratings for all exposure variables and occupational groups.

	Heavy back loading[a]	Lifting more than 25 kg[a]	Static, non-neutral trunk posture[a]	Extreme trunk postures (flexion or extension)[a]	Whole-body vibration[b]
Asphalt workers	2	1	1	1	3
Brick layers	3	3	2	3	1
Concrete workers	3	3	2	3	1
Crane operators	1	1	1	1	1
Drivers	2	2	1	1	2
Electricians	2	1	2	2	1
Floor layers	2	3	3	3	1
Foremen	Ref	Ref	Ref	Ref	Ref
Glass workers	2	2	1	2	1
Heavy machinery operators	1	1	1	1	3
Insulators	2	1	2	2	1
Painters	2	1	2	2	1
Plumbers	2	2	2	3	1
Preparatory workers	2	2	2	2	2
Refrigerator technicians	2	2	2	2	1
Repairers	2	2	2	2	1
Rock workers	3	2	2	2	1
Roofers	3	3	3	3	1
Sheet-metal workers	2	2	2	2	1
White-collar workers	Ref	Ref	Ref	Ref	Ref
Wood workers	2	3	2	2	1

[a] frequency: 1 = low, 2 = moderate, 3 = high
[b] 1 = none 2= acceptable, 3 =high

Table S2. Relative risk of surgically treated LDH by occupational group for total study cohort (occupational groups listed in descending order of RR). Poisson regression models adjusted for age, height, BMI, smoking, and date of surgery.

	n	Person-years	Cases	IR	RR	95% CI
White-collar workers	9 703	185 144	55	29.71	1.00	1
Crane operators	2 987	54 465	29	53.24	1.81	1.15-2.84
Roofers	1 201	28 928	16	55.31	1.72	0.99-3.01
Refrigerator technicians	1 191	31 166	18	57.76	1.72	1.01-2.93
Plumbers	21 884	506 392	257	50.75	1.61	1.20-2.16
Concrete workers	27 587	548 571	268	48.85	1.56	1.16-2.08
Floor layers	4 910	122 674	60	48.91	1.54	1.07-2.23
Brick layers	8 138	174 906	78	44.60	1.44	1.02-2.04
Heavy machinery operators	9 879	211 084	91	43.11	1.37	0.98-1.92
Wood workers	57 632	1 425 410	620	43.50	1.37	1.04-1.80
Insulators	2 500	61 291	26	42.42	1.29	0.81-2.07
Repairers	2 421	52 296	21	40.16	1.29	0.78-2.13
Painters	20 637	504 839	195	38.63	1.24	0.92-1.68
Preparatory workers	9 817	217 027	86	39.63	1.23	0.88-1.73
Sheet-metal workers	10 959	282 800	102	36.07	1.12	0.81-1.56
Asphalt workers	3 590	80 153	28	34.93	1.08	0.68-1.70
Drivers	3 869	77 700	26	33.46	1.07	0.67-1.70
Electricians	33 884	894 152	289	32.32	1.01	0.75-1.35
Glass workers	2 480	62 196	20	32.16	1.00	0.60-1.67
Foremen	24 911	513 369	153	29.80	0.97	0.71-1.32
Rock workers	2 670	45 852	13	28.35	0.93	0.51-1.71

N – number workers; IR – incidence rate per 100,000 person-years; RR – relative risk; CI – confidence interval

Table S3. Comparative analysis - Biomechanical exposure factors and relative risk for case definition *hospitalization due to LDH* (N= 262 849, incl. 3 172 cases) in male construction workers. Poisson regression models adjusted for age, height, BMI, smoking, and date of hospitalization. JEM terms, except WBV, are for frequency of time spent working in the listed postures or under the listed loads.

	n	Person-years	Cases	IR	RR	95% CI
Heavy back loading						
Reference	34 614	697 840.3	272	38.98	1.00	1
Low	12 865	264 909.5	162	61.15	1.54	1.26-1.87
Moderate	175 774	4 310 310.9	2259	52.41	1.35	1.19-1.54
High	39 596	796 706.9	479	60.12	1.55	1.33-1.79
Lifting more than 25 kg						
Reference	34 614	697 840.33	272	38.98	1.00	1.
Low	52 839	1 298 788.88	617	47.51	1.22	1.06-1.41
Moderate	174 195	4 044 313.90	2261	55.91	1.45	1.28-1.65
High	1 201	28 824.54	22	76.32	1.93	1.25-2.99
Static, non-neutral trunk posture						
Reference	34 614	697 840.3	272	38.98	1.00	1
Low	22 804	484 434.6	272	56.15	1.41	1.19-1.67
Moderate	199 320	4 736 355.5	2525	53.31	1.38	1.22-1.57
High	6 111	151 137.2	103	68.15	1.77	1.41-2.22
Extreme trunk postures (flexion or extension)						
Reference	34 614	697 840.3	272	38.98	1.00	1
Low	20 324	422 305.6	246	58.25	1.46	1.23-1.74
Moderate	144 191	3 570 825.2	1807	50.60	1.31	1.15.1.49
High	63 720	1 378 796.5	847	61.43	1.59	1.38-1.82
Whole-body vibration						
Reference	34 614	697 840.3	272	38.98	1.00	1
Low	201 081	4 787 429.3	2573	53.74	1.40	1.23-1.58
Moderate	13 686	293 984.1	165	56.13	1.41	1.16-1.71
High	13 468	290 513.9	162	55.76	1.39	1.14-1.69

n – number of workers; IR – incidence rate per 100,000 person-years; RR – relative risk; CI – confidence interval

Table S4. Comparative analysis - Self-reported exposure and pain ratings and the relative risk for case definition *hospitalization due to LDH* in the sub-group of the study cohort (N= 69 459 including 673 cases) who provided self-reported exposure and/or pain level for at least one of the following questions on health examination (1989-1993). Poisson regression models adjusted for age, height, BMI, smoking and date of surgery.

	N	Person-years	Cases	IR	RR	95% CI
Frequency of heavy lifting						
Rarely	9 491	223 701.8	67	29.95	1.00	1
Quite rarely	5 653	146 205.9	52	35.57	1.14	0.79-1.64
Sometimes	23 718	627 451.6	208	33.15	1.05	0.79-1.38
Fairly often	18 861	514 140.0	244	47.46	1.45	1.10-1.90
Often	11 736	318 649.4	169	53.04	1.59	1.20-2.11
Frequency of forward-bending or twisted working postures						
Rarely	8 558	205 142.1	65	31.69	1.00	1
Quite rarely	5 409	144 600.1	55	38.04	1.14	0.80-1.64
Sometimes	17 946	480 155.3	174	36.24	1.08	0.81-1.44
Fairly often	20 015	536 629.0	226	42.11	1.23	0.93-1.62
Often	17 525	462 524.8	221	47.78	1.39	1.05-1.83
Frequency of LBP during the last 12 months						
Rarely	16 286	444 398.9	105	23.63	1.00	1
Quite rarely	10 343	276 780.2	72	26.01	1.10	0.82-1.49
Sometimes	24 713	651 279.0	213	32.70	1.39	1.10-1.75
Fairly often	10 859	279 069.5	174	62.35	2.64	2.07-3.36
Often	6 897	165 827.5	167	100.71	4.47	3.50-5.71

N – number workers; IR – incidence rate per 100,000 person-years; RR – relative risk; CI – confidence interval
